# Comparing Visual Scoring of Lung Injury with a Quantifying AI-Based Scoring in Patients with COVID-19

**DOI:** 10.5334/jbsr.2330

**Published:** 2021-04-05

**Authors:** Charlotte Biebau, Adriana Dubbeldam, Lesley Cockmartin, Walter Coudyze, Johan Coolen, Johny Verschakelen, Walter De Wever

**Affiliations:** 1 University Hospitals Leuven, BE

**Keywords:** COVID-19, severity index, visual scoring, lung, artificial intelligence software

## Abstract

**Objectives:**

Fast diagnosis of Coronavirus Disease 2019 (COVID-19), and the detection of high-risk patients are crucial but challenging in the pandemic outbreak. The aim of this study was to evaluate if deep learning-based software correlates well with the generally accepted visual-based scoring for quantification of the lung injury to help radiologist in triage and monitoring of COVID-19 patients.

**Materials and methods:**

In this retrospective study, the lobar analysis of lung opacities (% opacities) by means of a prototype deep learning artificial intelligence (AI)-based software was compared to visual scoring. The visual scoring system used five categories (0: 0%, 1: 0–5%, 2: 5–25%, 3: 25–50%, 4: 50–75% and 5: >75% involvement). The total visual lung injury was obtained by the sum of the estimated grade of involvement of each lobe and divided by five.

**Results:**

The dataset consisted of 182 consecutive confirmed COVID-19 positive patients with a median age of 65 ± 16 years, including 110 (60%) men and 72 (40%) women. There was a correlation coefficient of 0.89 (*p* < 0.001) between the visual and the AI-based estimates of the severity of lung injury.

**Conclusion:**

The study indicates a very good correlation between the visual scoring and AI-based estimates of lung injury in COVID-19.

## Introduction

The recent Coronavirus Disease 2019 (COVID-19) pandemic outbreak, caused by infection with the highly contagious severe acute respiratory syndrome coronavirus 2 (SARS-CoV-2), has provoked worldwide quick responses [1]. Studies reported that the extent of ground-glass opacities (GGO) and consolidations on chest computed tomography (CT), as well as the presence of crazy paving are significant predictors for a more severe course of the disease or worse patient outcome [1,2]. As these CT findings allow an automatic machine quantification, artificial intelligence (AI) companies promptly developed automatic and accurate detection and quantification software for COVID-19 pneumonia [3,4,5,6,7,8,9]. Currently, some deep learning-based algorithms can accurately diagnose COVID-19 pneumonia with or without adjuvant clinical information [4,5,6,7,8,9]. However, in our institution we only used that software solution for the quick quantification of lung injury.

It is a common practice for radiologists to evaluate the pneumonia severity semi-quantitatively by visual scoring. However, this may be time consuming and subjective, so that its validity depends on the radiologists’ experience [4]. Thus, AI-based software can provide a more reproducible solution for the full assessment of lung injury.

In this retrospective study, we have analysed CT images of 182 patients who underwent a non-contrast chest CT and had a recently confirmed diagnosis of COVID-19 by Reverse Transcriptase Polymerase Chain Reaction (RT-PCR). The aim of this study was to evaluate if the AI-based software estimates correlate with a visual scoring system for the quantification of the lung injury.

## Material and Methods

### Subjects

The retrospective study was approved by the ethics committees, and written informed consent was waived by the Institutional Review Board. Between March 21, 2020, and April 11, 2020, 763 patients underwent RT-PCR on nasopharyngeal swab for COVID-19 together with a non-contrast chest CT. The combination of both examinations was exclusively reserved for earlier proven COVID-19 patients with worsening respiratory status or for medical triage of patients with suspected COVID-19 who present with moderate to severe clinical features and a high pre-test probability of COVID-19 pneumonia according to the Fleischner Society Statement on Chest Imaging and COVID-19 [10]. Those finally diagnosed with COVID-19 infection by RT-PCR on respiratory specimens were retained for this study. The chest CT was acquired prior to or within a time interval of four days of the RT-PCR test.

### Chest CT scan parameters

All CT examinations were performed on a 128 detector-row CT scanner (Siemens Definition Flash) with a single breath hold using the same scan parameters: gantry speed of 0.5s per rotation, slice collimation: 128 × 0.6 mm, pitch factor 1.2, slice thickness 1 mm and 3 mm, slice increment 0.7 mm and 3 mm, except for mAs and kV settings that were depending on patient weight (<50 kg: 80 kV and 30 mAs; 50–80 kg: 120 kV and 20 mAs; >80 kg: 140 kV and 28 mAs).

### Evaluation of severity

**Figure 1:**
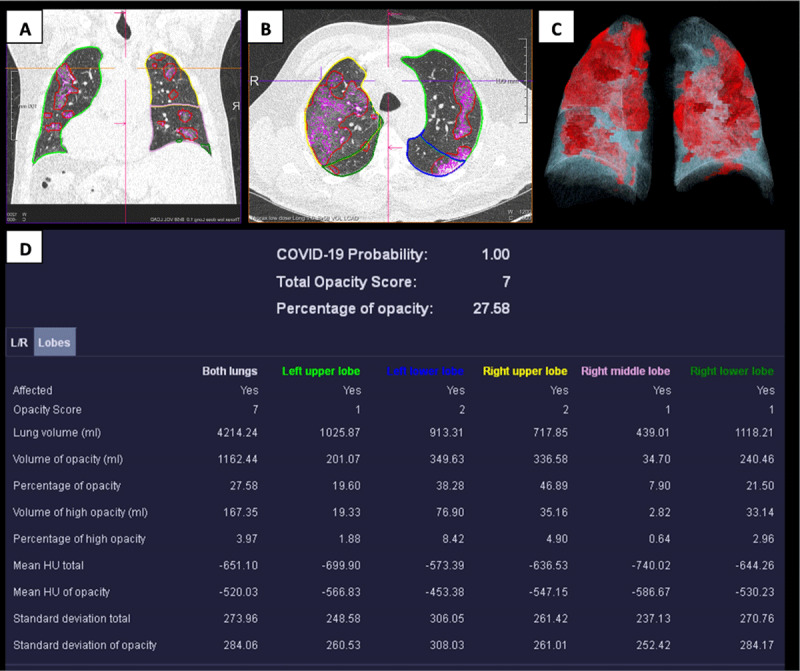
Coronal **(A)** and axial **(B)** lung reconstructed CT images with delineation of the lung lobes, fissures, and the opacities performed by software analysis. **(C)** A 3D view of the lung opacities (in red colour). **(D)** An overview of the absolute and relative lung involvement per lung or lung lobe, the lung volume as well as a probability index for COVID-19.

The severity of lung injury was assessed qualitatively and quantitatively using a severity index. The qualitative severity score was based on a visual grading of the lung injury per lobe into six categories (0: no involvement, 1: 0–5% involvement, 2: 5–25% involvement, 3: 25–50% involvement, 4: 50–75% involvement, 5: >75% involvement), and was performed by a single radiologist (a final year resident in radiology with great interest in thoracic imaging) and approved by a >20-year experienced thoracic radiologist. The quantitative severity score was based on a prototype deep learning algorithm, that is, Syngovia® CT Pneumonia Analysis of which the permission was granted (Siemens HealthCare, Forchheim, Germany) [11]. This software performs an automated segmentation of the lung parenchyma and analyses the lung opacities on CT (*https://store.teamplay.siemens.com/api/download/media/Siemens%20Healthcare%20GmbH/CT%20Pneumonia%20Analysis/1.0/manual.pdf*). This results in multiplanar reformation series overlaid with delineations of the opacities in the lungs. Absolute and relative volumes and mean Hounsfield Units (HU) of the opacities are provided per lung and per lung lobe (***Figure 1***). The segmentation of the lung opacities has a multicentred built-in training process that is continuously improving. Consolidations were defined as opacities of -200 HU or more. All segmentation results derived from the algorithm were visually evaluated and corrected slice-by-slice by the same radiologist. Tumoral processes in the lungs were therefore manually excluded from the analysis.

### Statistical analysis

Continuous variables are given as mean ± SD. The Spearman’s correlation coefficient (*r*_s_) measures the strength and the direction of association between two ranked variables (visual scoring versus deep learning-based scoring). Correlation of the total lung opacity volume assessed by the algorithm and the visual scores was performed by calculating the ratio of the sum of the visual scores of each lobe to the sum of the maximum score (5 × 5 = 25) referring to the total lung involvement. The analysis was performed by the IBM Statistical Package for Social Sciences software (SPSS version 13, IBM Corp., Armonk, NY, USA).

## Results

Of the 763 patients who underwent CT and RT-PCR, 182 (23.9%) were diagnosed with COVID-19 (***Table 1***). The average patient age was 65 ± 16 years and there were 110 (60%) men and 72 (40%) women.

**Table 1:** Summary of Patient Characteristics (n = 182).


*PARAMETER*	*VALUE; N(%)*

Sex	

Men	110 (60.4)

Women	72 (39.6)

Age (y)	

Mean	65

Standard deviation	16.22

Range	22–91

Body mass index (kg/m^2^)	

Mean	27.4

Standard deviation	0.47

Range	10.8–47.1


The qualitative visual grading scores and the quantitative severity index were assessed for all patients and for each lung lobe individually. ***Table 2*** shows the absolute (and relative) number of ratings of each visual score and the absolute and relative volume of lung opacity determined by the AI-based algorithm. In both scoring systems the lower lobes had a higher grade of involvement followed by the upper lobes. The right middle lobe was the less affected lobe.

**Table 2:** Lung involvement severity index.


	LEFT UPPER LOBE N(%)	LEFT LOWER LOBE N(%)	RIGHT UPPER LOBE N(%)	RIGHT MIDDLE LOBE N(%)	RIGHT LOWER LOBE N(%)	TOTAL LUNG VOLUME (ML)	VOLUME OPACITIES (ML)	OPACITY (%)	HIGH OPACITY (%)

**Pneumonia Analysis software:**									

Mean	10.19	17.80	13.04	9.49	19.57	4142.08	492.82	13.37	3.10

SDD	15.60	20.08	20.42	16.52	22.07	1256.66	502.83	15.08	4.60

Range	0–74.32	0–84.95	0–100	0–84	0–94.08	1691.97–8179.75	0.05–2820.67	0–82.23	0–29.61

**Visual scoring:**									

0: 0%	22(12.1)	6(3.3)	22(12.1)	35(19.2)	9(4.9)				

1: 0–5%	77(42.3)	58(31.9)	80(44.0)	80(44.0)	53(29.1)				

2: 5–25%	49(26.9)	62(34.1)	41(22.5)	40(22.0)	60(33.0)				

3: 25–50%	26(14.3)	41(22.5)	24(13.2)	21(11.5)	42(23.1)				

4: 50–75%	8(4.4)	11(6.0)	11(6.0)	4(2.2)	12(6.6)				

5: 75–100%	0(0.0)	4(2.2)	4(2.2)	2(1.1)	6(3.3)				


**Figure 2:**
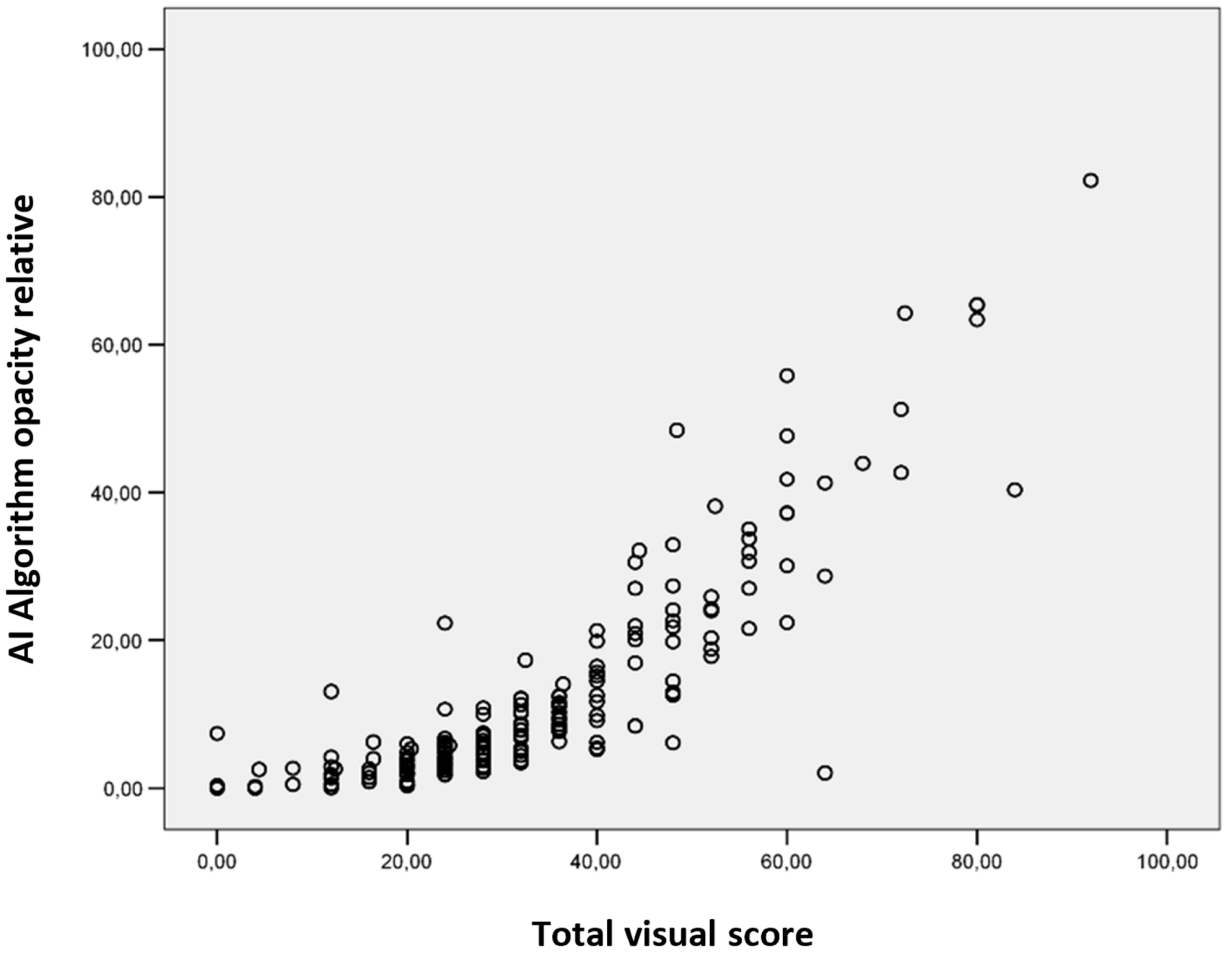
Relative volume of total lung opacity as a function of the visual scoring assessment, illustrating a significant monotonic increasing relation between the qualitative and quantitative scores of lung opacities.

**Figure 3:**
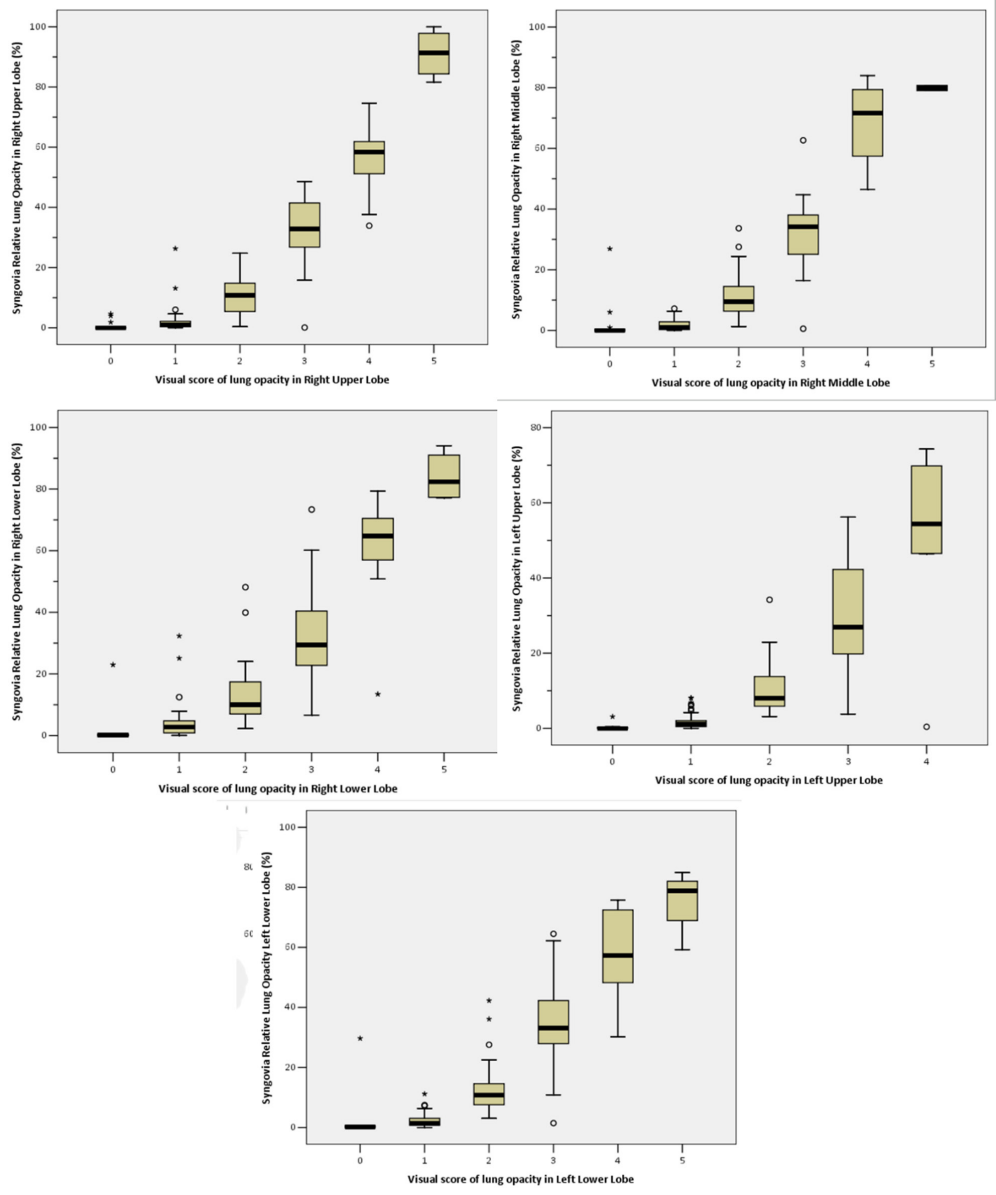
Relative volume of total lung opacity as a function of the visual score for the lung opacity, illustrating a monotonic increasing relation between the qualitative and quantitative scores of lung opacities for each lung lobe separately.

***Figure 2*** shows the AI-based software assessment of the relative lung opacity as a function of the relative sum of visual scores for all lung lobes, illustrating a monotonic increasing relation between both. The Spearman correlation coefficient *r*_s_ was 0.89 (*p* < 0.001), indicating a very good correlation. When examining the correlation between both methods for each lung lobe separately, *r*_s_ values of 0.87, 0.85, 0.87, 0.88 and 0.89 were found respectively for the right upper lobe, right middle lobe, right lower lobe, left upper lobe and left lower lobe (all *p-values* < 0.001) (***Figure 3***).

## Discussion

Our study showed a very good correlation between the visual scoring and the AI-based scoring in the assessment of the total lung involvement in COVID-19 pneumonia. The distribution of lung involvement was also consistent with earlier studies, confirming the predominant basal distribution of COVID-19 pneumonia [3,12]. Most studies involving automated solutions for chest CT were mainly developed to predict the presence of COVID-19 via a binary response. We found only two other studies where lung severity in COVID-19 was assessed as well [3,4]. Chaganti et al. used deep learning to automatically compute the percentage of opacity and lung severity score by segmenting ground glass opacities, consolidations, and lung (lobes) in COVID-19 patients [3]. The ground truth was established by computing the same measures from manual annotations of the lesions and lung (lobes). The Pearson correlation coefficient between the algorithm and manually defined opacities was 0.92 for all opacities, 0.97 considering only high opacities (consolidations defined as –200 HU or more) (all *p-values* < 0.001). Similar correlations were obtained in the study of Lessmann et al. using CORADS-AI to score the extent of pulmonary COVID-19 infection on chest CT [4]. CORADS-AI consists of three deep learning algorithms that automatically segment the pulmonary lobes, assign a CORADS score for the suspicion of COVID-19, and assign a CT severity score for the degree of parenchymal involvement per lobe [4]. This was compared to the visual scoring of eight independent human observers who described semi-quantitatively the extent of parenchymal involvement per lobe using a predefined 6-point scale [4].

Some earlier studies have shown that human readings tend to overestimate the extent of disease [4]. However, AI can help to make an accurately, quantifiable, and reliable assessment of the pneumonia severity, allowing disease monitoring. The inverse is also true, as the study of Lessmann et al. demonstrated that four out of 108 automatic measurements were overestimated based on severe motion artifacts or aspiration pneumonia, underlining the importance of verification of automatically determined severity scores by human reading [4].

For prognostic analysis, Huang et al. used a deep-learning method to quantitatively evaluate the severity of COVID-19 [5]. They demonstrated a significant difference in lung opacification percentage among patients with different clinical severity [5]. Most of the published studies were using the diagnostic properties of AI-software and all showed good diagnostic values for COVID-19 pneumonia [4,5,6,7,8,9]. Meanwhile there is still a lot of work to be done for pattern recognition, since in COVID-19 pneumonia next to the typical and common CT findings (e.g., GGO, consolidations, crazy paving), there may be atypical (e.g., enlarged lymph nodes, pleural effusion, tree-in-bud pattern) or rare (e.g., reversed halo sign, cysts, bronchiectasis) findings [1]. There are also overlaps between the CT characteristics of different lung infections/diseases (e.g., other viral pneumonias such as H1N1 influenza, cytomegalovirus pneumonia, or atypical pneumonia) [1]. As mentioned by Laghi A et al. [13], several limitations in the diagnostic analysis of COVID-19 on chest CT must be kept in mind: First, approximately 50% of patients with COVID-19 infection have a normal CT scan if scanned early after the onset of symptoms [13,14]. Second, there are no pathognomonic CT findings of COVID-19 infection and they substantially overlap with other diseases [13]. Third, the CT findings are evolutive and different CT characteristics may be found during the course of the disease [12,13]. In addition, the response to the lung infection seems to be dependent on age, immune status, and underlying comorbidity [6]. Finally, most of the studies had important selection bias, with patients with limited pre-existing lung disease and originating from regions with high prevalence of COVID-19 and low prevalence of seasonal influenza and respiratory syncytial virus infections. Ultimately, AI systems need to be trained with larger datasets before they can be expected to correctly interpret studies with overlapping abnormalities due to other types of pneumonia or other diseases (e.g., congestive heart failure, pulmonary fibrosis, or acute respiratory distress syndrome).

## Limitations

There are some limitations in our study. First, there are too big intervals in the visual scoring system. For example, a patient with an automatic scoring of 24% can be visually scored in classification 2 (5–25%) or 3 (25–50%), but the scoring in class 3 reduces the performance of the AI-based software. Second, our study is based on a study cohort of 182 patient (small sample size). However, the deep-learning-based software is self-learning by corrections made by radiologists from multiple institutions.

## Conclusion

Artificial intelligence is a useful tool in determining the extent of lung involvement in COVID-19 during the pandemic outbreak, thus facilitating triage and providing a prognostic value on a patient basis. It is likely that the development of AI models integrating clinical and biological information can further augment radiologists’ performance to distinguish COVID-19 from other pneumonias and improve the diagnostic in difficult cases (early phase and late phases).
